# Living-donor Lobar Lung Transplantation - Initiation and Development - Secondary Publication

**DOI:** 10.31662/jmaj.2023-0207

**Published:** 2024-02-09

**Authors:** Hiroshi Date

**Affiliations:** 1 Department of Thoracic Surgery, Kyoto University Graduate School of Medicine, Kyoto, Japan

**Keywords:** living-donor lobar lung transplantation, cadaveric lung transplantation, right-to-left inverted transplantation, upper lobe-preserving lung transplantation

## Abstract

Due to the difficulty of finding brain-dead donors, in October 1998, I performed the first living-donor lobar lung transplantation (LDLLT) for a ventilator-dependent 24-year-old female patient with bronchiectasis using lobes from her parents. The patient is still alive and in good health 25 years after the transplantation. Over time, the indications for LDLLT have expanded to include pulmonary hypertension, pulmonary fibrosis, congenital genetic diseases, pulmonary complications after stem cell transplantation, and, more recently, severe lung injury due to COVID-19 infection. In 2022, we successfully performed an ABO-incompatible LDLLT. To address size mismatches, we have developed new LDLLT techniques, such as right-to-left inverted transplantation, upper lobe-sparing transplantation, and segmental lung transplantation. Our published studies cover a range of topics, encompassing both basic and clinical research. Of particular significance is the observation that LDLLT offers immunological advantages over cadaveric lung transplantation (CLT). Having conducted 353 cases of lung transplantation, including 161 LDLLTs and 192 CLTs, our 5-year survival rates were 83% after LDLLT and 74% after CLT, surpassing the 55% 5-year survival rate reported by the International Society for Heart and Lung Transplantation. We have hosted numerous observers and research fellows from 15 countries. In addition, I have contributed to LDLLT procedures not only in Japan but also abroad. It brings me great satisfaction to think that my educational efforts may ultimately lead to saving the lives of those suffering from end-stage respiratory failure around the world.

## I. The Journey to Introducing Living-donor Lobar Lung Transplantation (LDLLT)

The University of Toronto group, led by Dr. Joel D. Cooper, performed the first successful human lung transplantation for a patient with idiopathic pulmonary fibrosis in 1983. Subsequently, the same group successfully performed bilateral lung transplantation in 1986. Because of their pioneering work, lung transplantation has been initiated in many institutions worldwide and has been accepted as a life-saving procedure for patients with end-stage respiratory failure.

In 1988, Dr. Cooper moved from Toronto to Washington University in St. Louis and established a new lung transplant program. I was fortunate to become a research fellow in his laboratory in 1989. During my time there, I conducted various animal research studies, including 24-hour lung preservation, and observed human lung transplantation procedures, which were performed almost every week. I was deeply impressed by the positive impact of lung transplantation, as I witnessed patients’ miraculous recoveries following highly sophisticated surgical procedures. This experience led me to dedicate my career to lung transplantation.

I returned to Japan in 1991, obtained the Educational Commission for Foreign Medical Graduates certification, and returned to the USA in 1993 to pursue a clinical transplant fellowship. During this second stay in the USA, I participated in 35 lung transplantation cases. In 1995, I returned to Japan with a strong desire to introduce lung transplantation to the country. However, it was a challenging time for organ transplantation in Japan. Since Dr. Wada’s human heart transplantation in 1968, no thoracic organ transplants have been performed. Brain death was not legally accepted as a death in Japan. However, in 1997, a transplant law was finally enacted, and brain death was recognized as a valid criterion for death. The law was quite stringent, requiring potential donors to carry a donation card with their signature. Consequently, no brain-dead donors became available for the next two years.

I returned to Japan from the USA in 1995 and began preparing for a new lung transplant program at Okayama University. However, it did not materialize for 3 years because of the lack of brain-dead donors. I was frustrated and deeply sorry for the patients who passed away without the opportunity for a lung transplant. This led me to conceive the idea of LDLLT, a procedure pioneered by the University of Southern California group in the early 1990s to address the shortage of brain-dead donors.

**Figure 1.**
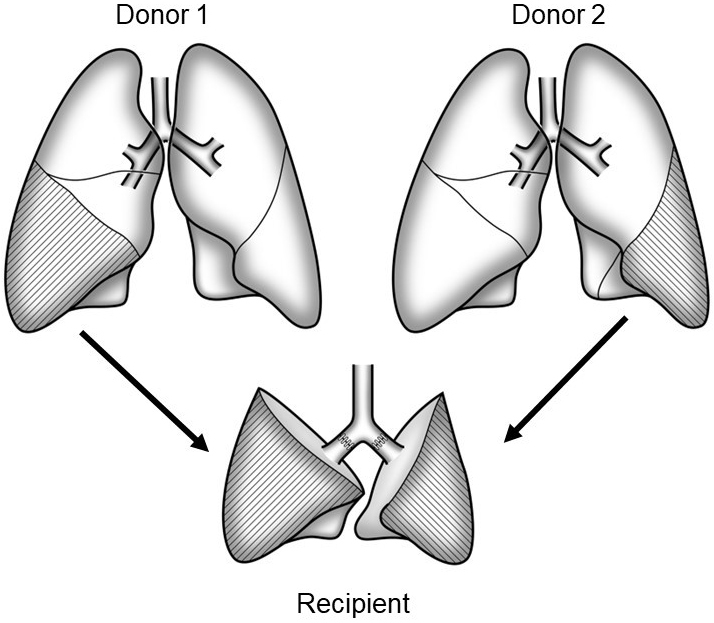
Bilateral living-donor lobar lung transplantation Two healthy donors donate either the right or left lower lobes, which are transplanted into the recipient as a whole right and left lung.

In LDLLT, two healthy donors each donate either their right or left lower lobes, and these two lobes are implanted as a whole right and left lung into the recipient (Figure 1). Because the transplanted grafts are relatively small, LDLLT was initially indicated for pediatric patients who received lobes from their parents. The lung is a nonregenerating organ, and lung donors permanently lose their pulmonary function. In the case of kidney and liver transplants, only one living-donor is required; whereas in LDLLT, two living donors are needed. Despite these challenging factors, I embarked on the preparation of an LDLLT program because it was the only practical option.

## II. The First Successful LDLLT

In October 1998, I received a consultation from Shinshu University regarding a potential LDLLT for a 24-year-old female patient using lobes from her parents ^(1)^. The patient was already on a ventilator due to a severe Pseudomonas infection. Her father had an ABO-incompatible blood type; therefore, we explored the possibility of using lobes from her younger sister and her mother. Because the patient was an adult, the estimated graft vital capacity was only 51.7% of her predicted vital capacity. I believe that the success rate of LDLLT would be approximately 70%.

**Figure 2.**
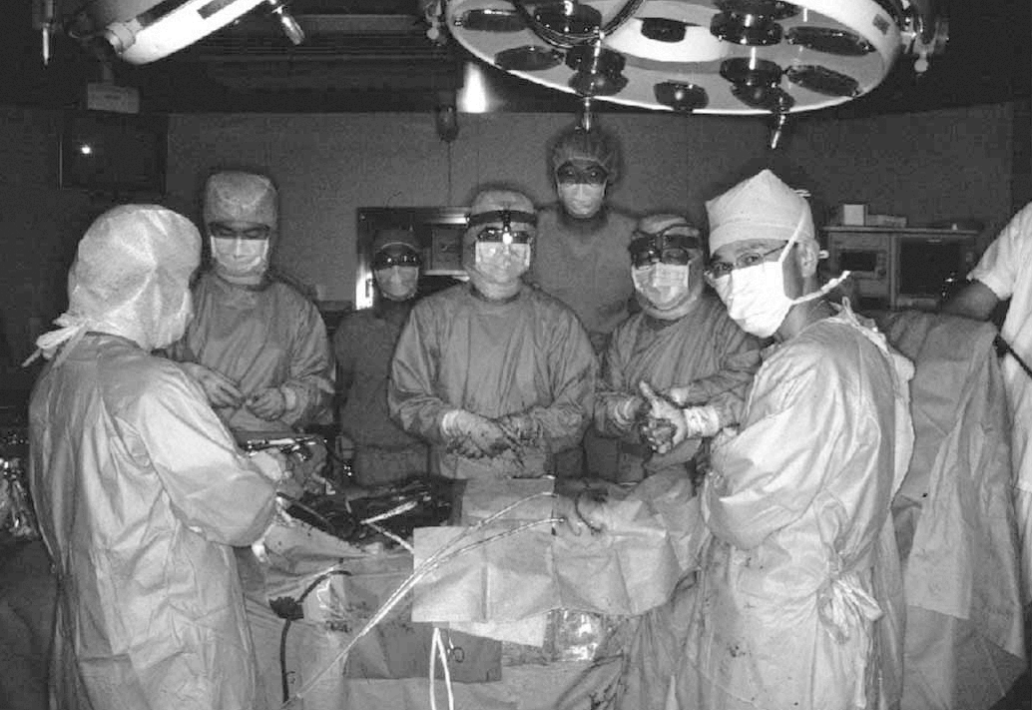
First living-donor lobar lung transplantation at Okayama University (October 28, 1998) The author is situated in the center.

After 30 years of no thoracic organ transplantation, we performed the first LDLLT on October 28, 1998, with significant attention from the mass media. The patient’s condition rapidly deteriorated on the morning of the transplantation. Her PaCO2 exceeded 150 mmHg when she was brought to the operating room. At 10:49 am, I began the surgery with a sense of trepidation. It was challenging to remove the infected lungs due to severe pleural adhesions. The bleeding exceeded 2 L. Eventually, I successfully removed the lungs and implanted the small grafts. When the implanted lobes were reperfused and reventilated, the appearance of healthy, pink lobes indicated that they were functioning well. The operation was successfully concluded at 6:35 p.m. after 7 hours and 46 minutes (Figure 2).

**Figure 3.**
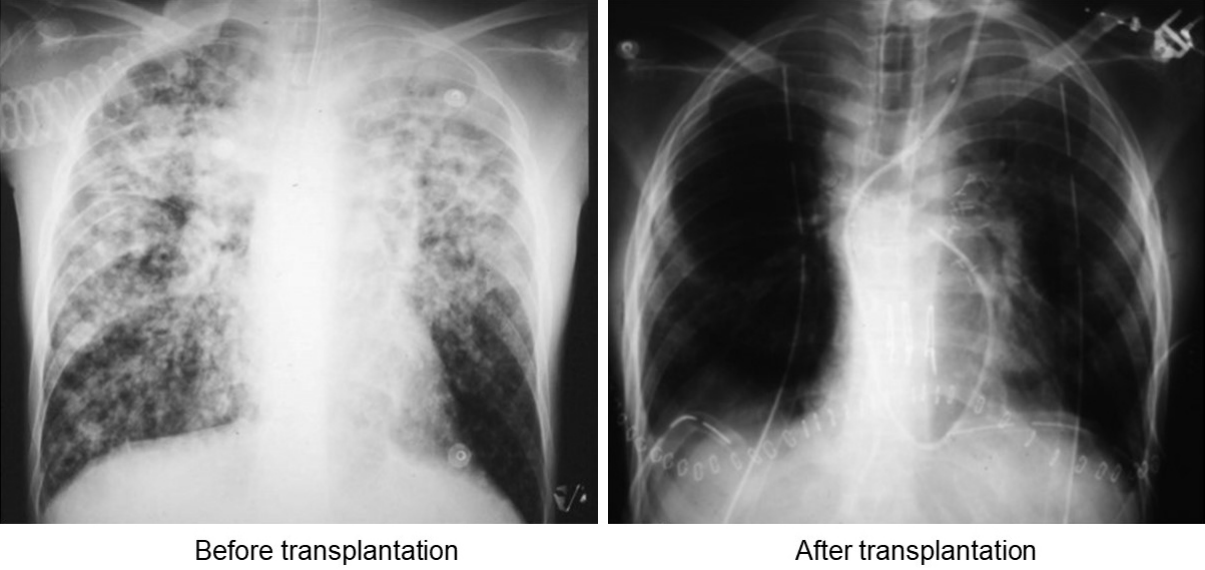
Chest X-ray before and after bilateral living-donor lobar lung transplantation Before transplantation, the patient required a ventilator due to Pseudomonas pneumonia. After transplantation, chest X-ray showed a significant improvement with clear translucency.

The patient was discharged two months after LDLLT with a clear chest X-ray (Figure 3). Remarkably, the patient is still alive and in good health 25 years after the transplantation.

## III. Expanded Indications for LDLLT

When LDLLT was first introduced in the USA, it was primarily indicated for pediatric patients with cystic fibrosis. Concerning this, I performed the first LDLLT procedure for a pediatric patient in March 2001. The patient was a 13-year-old boy suffering from bronchiolitis obliterans, a complication of Stevens-Johnson syndrome, and he was severely debilitated with marked hypercapnia ^(2)^. He underwent LDLLT using lobes donated by his parents and made a remarkable recovery, eventually returning to the level of playing basketball. I also successfully performed a single LDLLT for a 10-year-old boy with pulmonary hypertension, using his mother’s lobe.

One significant concern was whether adult patients with pulmonary hypertension who received LDLLT with relatively small grafts might develop primary graft failure. Given the promising outcomes reported for adult patients who received single lung transplantation from brain-dead donors, I expanded the eligibility for LDLLT to include adult patients with pulmonary hypertension. Our research demonstrated that LDLLT for pulmonary hypertension resulted in sustained decreases in pulmonary artery pressure and increased cardiac output over an extended period ^(3)^.

In 2007, after relocating from Okayama University to Kyoto University, I further broadened the range of indications for LDLLT to encompass conditions, such as pulmonary fibrosis, congenital genetic diseases, and pulmonary complications following hematopoietic stem cell transplantation ^(4)^. In 2021, we successfully performed the first LDLLT for a female patient who had suffered severe lung injury due to COVID-19 infection ^(5)^. She had been on extracopereal membrane oxygenation for three months, and her lungs were essentially nonfunctional. Following the transplantation of lobes from her husband and son, her chest X-ray showed a remarkable improvement. This success illuminated the potential of LDLLT to benefit patients with irreversible lung injury caused by COVID-19 infection. In 2022, we successfully performed an ABO-incompatible LDLLT. In ABO-incompatible transplantation, it is crucial to manage antibody-mediated rejection between the recipient’s anti-A/B antibodies in the serum and the A/B antigens in the endothelium of the donor grafts. Desensitization therapy has been effectively employed in living kidney and liver transplantation. However, concerns have been raised regarding whether desensitization therapy increases the risk of infection in lung transplantation. To mitigate this, we implemented desensitization using rituximab, immunosuppressants, and plasma exchange, along with the administration of γ-globulin and antibiotics. The first case was a 14-year-old patient who was dependent on a ventilator with blood type O. She received the right lower lobe from her father, who had blood type B, and the left lower lobe from her mother, who also had blood type O. The postoperative course was uneventful, and the patient has since resumed a normal life. In situations where critically ill patients cannot find ABO blood type-compatible donors within their family, ABO blood type-incompatible LDLLT is a valuable alternative.

## IV. Development of New Transplant Procedures in LDLLT

In a standard LDLLT, two lower lobes are typically obtained from healthy adult living donors and implanted. However, for adult recipients, two lower lobe grafts may be insufficient in size. To address this concern, we developed a technique that involves a right-to-left inversion, taking advantage of the fact that the right lower lobe is approximately 25% larger than the left lower lobe ^(6)^. Since all hilar structures are also inverted in this procedure, we conducted simulations of the anastomotic processes using a 3D model created from CT scans of both the donor and recipient. Furthermore, we developed an upper lobe-preserving technique ^(7)^.

However, the lower lobe of an adult lung may be excessively large for pediatric patients whose height is less than 100 cm. To address this problem, we have introduced single lobe transplant ^(8)^ and segmental lung transplant ^(9)^ procedures.

These new transplant techniques that we have developed encompass 22 right-to-left cases, 14 upper lobe-preserving cases, and 7 segmental transplant cases. They have proven to be life-saving options for patients who were otherwise not suitable candidates for LDLLT.

## V. Research on LDLLT

We have published various studies, including both basic and clinical research, covering topics such as changes in the recipient’s cardiac function, size matching based on 3D-CT and pulmonary function, alterations in the shape of the remaining lobe in living donors, and donor-specific antibodies ^(10)^. Among these, it is particularly noteworthy that LDLLT offers immunological advantages over cadaveric lung transplantation (CLT). It is well-established that the development of de novo donor-specific antibodies (dnDSA) is a predictor of poor outcomes. We were the first to demonstrate that dnDSA occurs less frequently and is detected significantly later in LDLLT than in CLT. In CLT, a recipient receives grafts from a single donor, whereas in LDLLT, grafts are obtained from two different donors. Our research has also indicated that the prognosis following chronic allograft dysfunction (CLAD) development is better in LDLLT because unilateral CLAD is more common, and the contralateral unaffected lung may continue to function normally ^(11)^.

## VI. Outcome of LDLLT

I conducted 353 cases of lung transplantation at Okayama and Kyoto Universities between October 1998 and August 2022. This experience encompasses 161 LDLLTs and 192 CLTs. Notably, my experience in LDLLT is the largest in the world. The 5-year survival rates were 83% after LDLLT and 74% after CLT, which exceeded the 5-year survival rate (55%) reported by the International Society for Heart and Lung Transplantation. There were no operation-related deaths in living-donor procedures, and all donors successfully resumed their previous lifestyles.

## VII. Promoting the Global Adoption of LDLLT

I attribute my success in lung transplantation to the education I received from Professor Cooper in the USA. Therefore, I have dedicated significant efforts to advancing education and promoting the global adoption of LDLLT. We have hosted numerous observers and research fellows from 15 countries, including Italy, Sweden, England, Ukraine, Egypt, UAE, Saudi Arabia, India, China, Korea, Taiwan, Hong Kong, Malaysia, the USA, and Chile. In addition, I have contributed to LDLLT procedures not only in Japan (Okayama, Kyoto, Fukuoka, Nagasaki, Chiba, and Tokyo) but also abroad, including Israel, China, and Korea.

It brings me great satisfaction to think that my educational efforts may ultimately lead to saving the lives of those suffering from end-stage respiratory failure around the world.

## Article Information

This article is based on the study, which received the Medical Award of The Japan Medical Association in 2022. This is a revised English version of the article originally published in Japanese in the Journal of the Japan Medical Association 2023;151(10):1833-6 ^(12)^. The original version is available at https://med.or.jp/cme/jjma/newmag/pdf/151101833.pdf. Only members of the Japan Medical Association are able to access it.

### Conflicts of Interest

The author received a lecture fee from Johnson and Johnson, and research funding from Taiho, Adachi, and Hogi Medical.
